# Machine learning and statistics to qualify environments through multi-traits in *Coffea arabica*

**DOI:** 10.1371/journal.pone.0245298

**Published:** 2021-01-12

**Authors:** Weverton Gomes da Costa, Ivan de Paiva Barbosa, Jacqueline Enequio de Souza, Cosme Damião Cruz, Moysés Nascimento, Antonio Carlos Baião de Oliveira

**Affiliations:** 1 Department of General Biology, Federal University of Viçosa, Viçosa, Minas Gerais, Brazil; 2 Department of Statistics, Federal University of Viçosa, Viçosa, Minas Gerais, Brazil; 3 Brazilian Agricultural Research Corporation (EMBRAPA), Viçosa, Minas Gerais, Brazil; Wroclaw University of Science and Technology, POLAND

## Abstract

Several factors such as genotype, environment, and post-harvest processing can affect the responses of important traits in the coffee production chain. Determining the influence of these factors is of great relevance, as they can be indicators of the characteristics of the coffee produced. The most efficient models choice to be applied should take into account the variety of information and the particularities of each biological material. This study was developed to evaluate statistical and machine learning models that would better discriminate environments through multi-traits of coffee genotypes and identify the main agronomic and beverage quality traits responsible for the variation of the environments. For that, 31 morpho-agronomic and post-harvest traits were evaluated, from field experiments installed in three municipalities in the Matas de Minas region, in the State of Minas Gerais, Brazil. Two types of post-harvest processing were evaluated: natural and pulped. The apparent error rate was estimated for each method. The Multilayer Perceptron and Radial Basis Function networks were able to discriminate the coffee samples in multi-environment more efficiently than the other methods, identifying differences in multi-traits responses according to the production sites and type of post-harvest processing. The local factors did not present specific traits that favored the severity of diseases and differentiated vegetative vigor. Sensory traits acidity and fragrance/aroma score also made little contribution to the discrimination process, indicating that acidity and fragrance/aroma are characteristic of coffee produced and all coffee samples evaluated are of the special type in the Mata of Minas region. The main traits responsible for the differentiation of production sites are plant height, fruit size, and bean production. The sensory trait "Body" is the main one to discriminate the form of post-harvest processing.

## Introduction

The search for specialty coffee is growing both in the Brazilian and worldwide markets. The production of good quality coffee depends on several factors. Among these, the genotype, environment, and post-harvest processing of coffee are described in the literature as fundamental factors for a better final quality product [1–3].

Brazil has a great diversity of coffees, environments, and technological levels of production. These different influences affect the responses of important traits manifested in the coffee produced. Among the several coffee-producing municipalities, those located in the state of Minas Gerais, obtain good results in coffee quality concourses, which guarantees in the market space and added value to the product [4]. A wide territorial extension and environmental variation in the state of Minas Gerais, with altitudes between 400 and 1650 meters, result in multi thermal and water conditions. These combinations, associated with the production system, interfere in the phenology of Arabica coffee and condition different classes of beverage quality [4, 5].

The price of coffee is related to the size of the bean, according to Cheng et al. [6] small beans of the same variety bring lower prices. However, grain size changes according to the environment [7, 8] and larger grains do not necessarily have better behavior quality. Additionally, environmental factors, such as shade and high altitude, can influence the quality of coffee [9, 10]. The taste of coffee is also very sensitive to environmental changes. An increase in positive attributes (appearance and preference) along with a decrease in negative attributes (bitterness and astringency) was found in coffee grown in the shade [7, 11, 12].

Consumers of high-quality coffee may prefer the genotype with species labeling (for example, Arabica) or the production environment (generally country) [6]. Thus, there are several demands and objectives for discrimination of certain aspects in the coffee production chain, such as the discrimination of environmental factors [13, 14], genotypes [15], disease infection levels [16], among others. For example, Barbosa et al. [14] performed the discrimination of environments by measuring isotopic variables to discriminate environments for the appellation of origin, since this has become a requirement of the international market. Katsuhama et al. [13] were able to discriminate environments based on NIR spectroscopy, to avoid recurring commercial frauds that generally occur in the coffee production chain.

Because of this wide variation, to which coffee activity can be submitted, it is important to identify the main plant traits that are most affected by these changes, since some genotypes may show better patterns in specific environments [2, 17, 18]. With a greater understanding of the response of agronomics and sensory traits of beverage quality in the face of environmental variation, it is possible to determine the production sites and post-harvest processing most appropriate for the production of a given coffee profile.

Data collected in plant breeding studies often present a multi-trait multi-environment structure. However, these data are rarely analyzed in a combined analysis. Although combined analysis is a better representation of reality, it requires more complex models [19]. Together with the discrimination of environments based on multiple traits, it is of great importance also to recognize which of traits have the greatest effect on this discrimination.

For that, statistical models can be used to discriminate genotypes according to their production variation. However, not all discrimination statistical models are efficient when there are non-linearly separable problems [20]. Also, some of these methodologies assume that the data must present a multivariate normal distribution and a homogeneous variance and covariance matrix [21–23]. These assumptions can often be unreal, as when working with discrete and continuous traits simultaneously [24]. In these cases, it is appropriate to employ methodologies whose results can be taken from a set of mixed explanatory traits (discrete and continuous), so that the researcher can make decisions in the face of a multivariate big data set.

Computational intelligence is widely consolidated in computing and engineering areas with a high potential to circumvent discriminatory problems [25]. Machine learning models are used for different purposes and areas several, such as classification trees, and their extensions [26–30], in addition to artificial neural networks (ANNs) [31–35]. Used as a classification method, ANNs have certain advantages, such as being non-parametric [36] and tolerant of data loss [37]. Just like ANNs, classification trees, and their refinements do not require assumptions about the model [38]. Also, classification trees are better classifiers than linear statistical methodologies, allowing for non-linearity of data and easier interpretation [39], as it provides information on which attributes are most important for prediction or classification [26, 27, 40, 41].

In this context, the present study proposes to evaluate the efficiency of statistical methods and machine learning models for discriminating environments through multi-traits (agronomic and sensorial) of coffee genotypes. Additionally, it was intended to identify the main traits responsible for the variation of genotypes according to production sites and post-harvest processing.

## Materials and methods

### Experimental data

The field experiments were installed in three municipalities located in the Matas de Minas region, in the state of Minas Gerais, Brazil: Senhora de Oliveira, Araponga, and Paula Cândido. The study was carried out on private land, the owner of the land gave permission to conduct the study on this site. No specific permissions were required for activities in these locations. There is mutual trust between researchers and producers, where the researchers provide raw materials for production and the producers provide the sites to evaluate the experiments. The field studies did not involve endangered or protected species.

The experiments consisted of a randomized complete block design, with three replications, and 50 plants per plot. The samples of fruits collected in the municipality of Araponga were divided into two parts according to the type of post-harvest processing (Pulped and Natural). Thus, these samples together with samples from the other two locations resulted in the combination of four groups of environmental factors. The particularities of each environment were described in Table 1.

**Table 1:** Aspects of environments valued in the Matas de Minas region, Minas Gerais, Brazil.

Environment	Altitude (m)	Location coordinates	Post-harvest process	Spacing (m)	Planting year
**Paula Cândido**	680	20° 48’ 52’’ S	Pulped	2.5 x 0.5	2012
		42° 58’ 37’’ W			
**Senhora de Oliveira**	910	20° 50’ 32’’ S	Pulped	2.8 x 0.7	2009
		43° 23’ 34’’ W			
**Araponga**	1100	20° 38’ 48’’ S	Pulped	2.5 x 0.7	2013
		42° 30’ 41’’ W			
**Araponga**	1100	20° 38’ 48’’ S	Natural	2.5 x 0.7	2013
		42° 30’ 41’’ W			

At each environment, ten cultivars and one elite progeny Arabica coffee trees were evaluated, with different levels of rust resistance. The field experiments were conducted according to standard technical recommendations for arabica coffee cultivation [42, 43], except for chemical rust control, which was not carried out.

Thirty-one morpho-agronomic and post-harvest traits were evaluated. The morpho-agronomic traits, evaluated before harvest, were: Vegetative vigor, fruits maturation cycle (AMC), fruits maturity uniformity (MU), mature fruit size (FS), severity of cercosporiosis (Cer) and rust (Rust), plant height (PH), top (TopD) and stem diameter (StemD); the post-harvest traits were: Production (Prod), defect percentage (DefP), defects, sieves (S19, S18, S17, S16, S15, S14), moca grains (MGr, MGr11, MGr10, MGr09), sieve bottom (SB); and sensory attributes—fragrance/aroma (FragAr), taste, acidity, body, clean cup, sweetness, uniformity, aftertaste (Aft), balance, and overall impression [44].

Vegetative vigor was assessed by assigning scores according to a visual scale that ranged from 1 to 10, in which the score of 1 corresponds to plants with reduced vegetative vigor and marked symptom of depletion, and 10 to plants with marked vegetative growth of the branches, productive and without apparent symptoms of nutritional deficiencies or diseases [45]. The MU of the fruits was classified as uniform, moderately uniform, moderately nonuniform, and nonuniform, by assigning scores for these attributes, ranging from 1 to 4, respectively. The AMC was classified as early, early to medium, medium, medium to late, or late, receiving scores from 1 to 5 for these attributes, respectively. Mature fruit size (FS) was classified as small, medium, or large, with grades of 1 to 3, respectively. The evaluations of AMC and FS were carried out before harvest, taking as reference the cultivar Catuaí Vermelho IAC 144, classified as medium for AMC and FS.

Severity of cercosporiosis (Cer) and rust (Rust) were carried out in the peak months of the disease in the field (between March and July), in individual plants of the useful plots with scores from 1 to 5 for immune, resistant plants, moderately resistant, moderately susceptible and susceptible, according to criteria recommended by Fazuoli [46]. PH was determined in meters, by measuring the main stem (orthotropic branch) from the ground level to the last apical point of the coffee tree. The TopD was evaluated together with the height, measured in meters, in the width of the largest projection of the skirt, in the transversal direction about the planting line of the coffee trees. And StemD was measured in centimeters at the height of the plant’s collection region (about 5 cm from the soil).

To evaluate the maximum potential of the behavior quality offered by the genotypes, the fruit samples were composed only of fruit in the cherry stage, since completely mature. The fruits of lower density, pips, and badly granulated were eliminated from the samples. One of the fruit samples from the Araponga environment was sent to the drying process (natural coffee) in sieves with an area of 1 m^2^, in a suspended terrarium built with stainless wire mesh (2 mm^2^ mesh) and 7 cm tall wooden sides. The second sample from the Araponga site, together with the samples collected at the Senhora de Oliveira and Paula Cândido sites, were peeled in a Pinhalense model DPM-02 n° 928 sample peeler, driven by a 0.5-hp liter electric motor. After peeling, remaining husk residues, grains broken during the operation, and eventually drilled were removed from the samples. Then, the samples of peeled coffee were packed in plastic buckets of 20 L capacity, for demucilation through natural fermentation, for 24 hours (pulped coffee). Posteriorly of the fermentation period, the grains in parchment were washed in clean water, rubbing them against each other manually, under running water, and spread in the sieves like afore-mentioned, similar to the natural coffee.

The production of processed coffee was evaluated in liters of “field coffee” (“café da roça”—coffee at all stages of maturity) per plot, between May to July. After that, conversion of the volume of coffee collected to bags ha^-1^ was performed by approximation of values, considering the average yield of 480L of “field coffee” for each 60 kg bag of green coffee, which corresponds to the average yield adopted in all regions. Thus, bean production was evaluated in 60-kg bags of processed coffee per hectare (bg ha^-1^). In the drying process, the grains were spread on the sieves for drying in full sun, until the grains reached 11% moisture (bu). After drying, natural coffee beans with the endocarp attached were kept in double-leaf Kraft paper bags for a rest period of 30 to 40 days, to standardize the moisture content of the grains. Posteriorly this period, the samples were processed and conditioned in impermeable plastic bags and sent for tests physical of grain and sensory quality of the drink.

The sieve classification was also carried out from 300g of samples. Flat coffee beans were classified by the percentage of retention in sieves from 14 to 19 (S19, S18, S17, S16, S15, S14). The coffees beans were classified in flat and mocas, evaluated as a percentage of retention of each sieve and with grains retained at the bottom of the sieve. Defects, intrinsic and extrinsic, were classified by the sum of the number of defects found in 300g of the sample, according to the Official Brazilian Classification Table [47]. DefP corresponded the number of defects was weighed and converted to the elimination percentage, according to [47]. The moca grains retained in the sieves were classified from 9 to 11 (MGr11, MGr10, MGr09) and the total percentage of moca grains (MGr). The grains retained at the bottom of the sieve (SB) were also considered.

Sensory analysis of the drink was performed by three tasters and according to the Specialty Coffee Association of America [44], using a methodology for sensory evaluation of specialty coffees [48]. In this assessment, scores were given, in the range of 0 to 10 points, for each attribute fragrance/aroma (FragAr), acidity, body, flavor, clean cup, sweetness, uniformity, Aftertaste (Aft), balance, and overall impression [44]. Special coffees were those that reached a total score of 80 points or more. The total score was made up of the sum of the points awarded to each of the mentioned attributes [44].

### Pattern recognition

Initially, the hypothesis was formulated that there would be differences between the environments that could be revealed, posteriori, by the formation of clusters based on information from individuals, whose variation between performances would be determined by the macro-differences of the environments. The individuals were grouped according to the Kohonen Self-Organizing Map (KSOM) machine learning technique.

KSOMs are an unsupervised learning neural network method that detects similarities between entry patterns through a competition process [25, 49, 50]. The value of each individual assessed for the 31 traits in each environment was used as an input. The number of neurons equal to the number of environments was adopted, that is, four neurons. To assess the possibility of discrimination in a linear way of individuals according to environments, the standardized average Euclidean distance was used and for the iterative process, the number of 1000 iterations was stipulated.

### Discrimination methodologies

In another approach, the hypothesis was formulated that there would be, a priori, differences between individuals provided by the differences between the four environments. The veracity of this hypothesis was evaluated through an Apparent Error Rate (AER).

#### Statistical linear discriminant analysis

This procedure was adopted with the assumption that the environment whose genotypes belong to is known information. Thus, the consistency of the discrimination of environments was verified using the linear discriminant analysis of Fisher [21] and Anderson [22], as described by [51].

#### Construction of the Multilayer Perceptron network (MLP)

For the construction of the Multilayer Perceptron network (MLP), the Levenberg-Marquardt algorithm of feed-forward propagation with Bayesian regularization (trainbr function in MATLAB) was used to avoid the overfitting problem [52]. The Matlab software makes it possible to train a network with varying numbers of neurons used in the hidden layers. At the end of the process, the network topology that shows the best performance for the criteria previously specified by the user is saved. Preliminary analyzes were performed and, from these analyzes, we found that the use of two hidden layers showed greater stability for a lower error rate and an amplitude between 5 and 40 neurons for each hidden layer were sufficient, and it was trained for 5000 epochs. The momentum and performance gradient vary according to the processing and the data set. Thus, they were defined as described by the trainbr algorithm. The data were normalized, so that the maximum was 1 and the minimum was 0. The Nguyen-Widrow algorithm (INITNW) [53] was utilized to initialize the weights. This algorithm picks small random values as initial weights of the neural network. The weights are then modified in such a manner that the region of interest is divided into small intervals [54].

**Fig 1:**
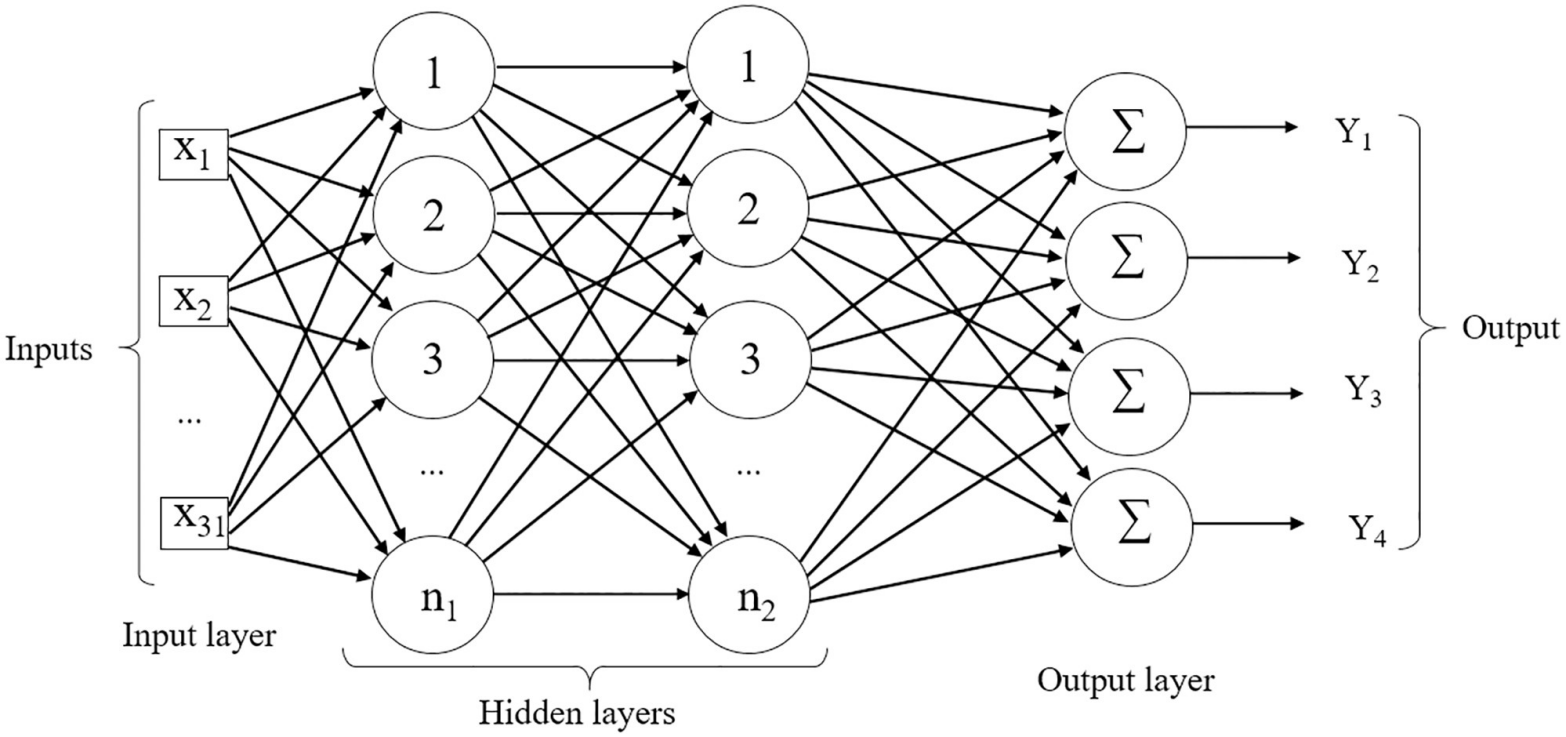
Example of an MLP Backpropagation network. Inputs X_1_ to X_31_ in the input layer refer to the 31 traits evaluated. Network architecture has two hidden layers and the number of neurons (n) ranging from five until 40 (n = 1, 2, …, 40). At the output, the network returns a matrix with values of 1 and 0 of dimension *i* x *j*, where *i* is the number of observations and *j* the number of environments.

The activation function used was the hyperbolic tangent sigmoid, $f(n)=\frac{2}{\left(1+e^{-2n}\right)}-1$, in which it assumes values between -1 and 1 (tansig function in MATLAB), to determine the form and intensity of change in the values transmitted from one neuron to another [55]. The model used in the Multilayer Perceptron network is shown in Fig 1.

#### Construction of the Radial Basis Function network (RBF)

The Radial Basis Function (RBF) is a type of network considered hybrid, that is, it presents the first stage unsupervised, to grouping the data and, later, a supervised step to capture the pattern obtained. In the first stage of training, the K-means method was used. The number of K groups was given by the number of environments (K = 1, 2, 3, and 4). In the second stage, the training is carried out similarly to that performed in the MLP networks, that is, supervised [56].

**Fig 2:**
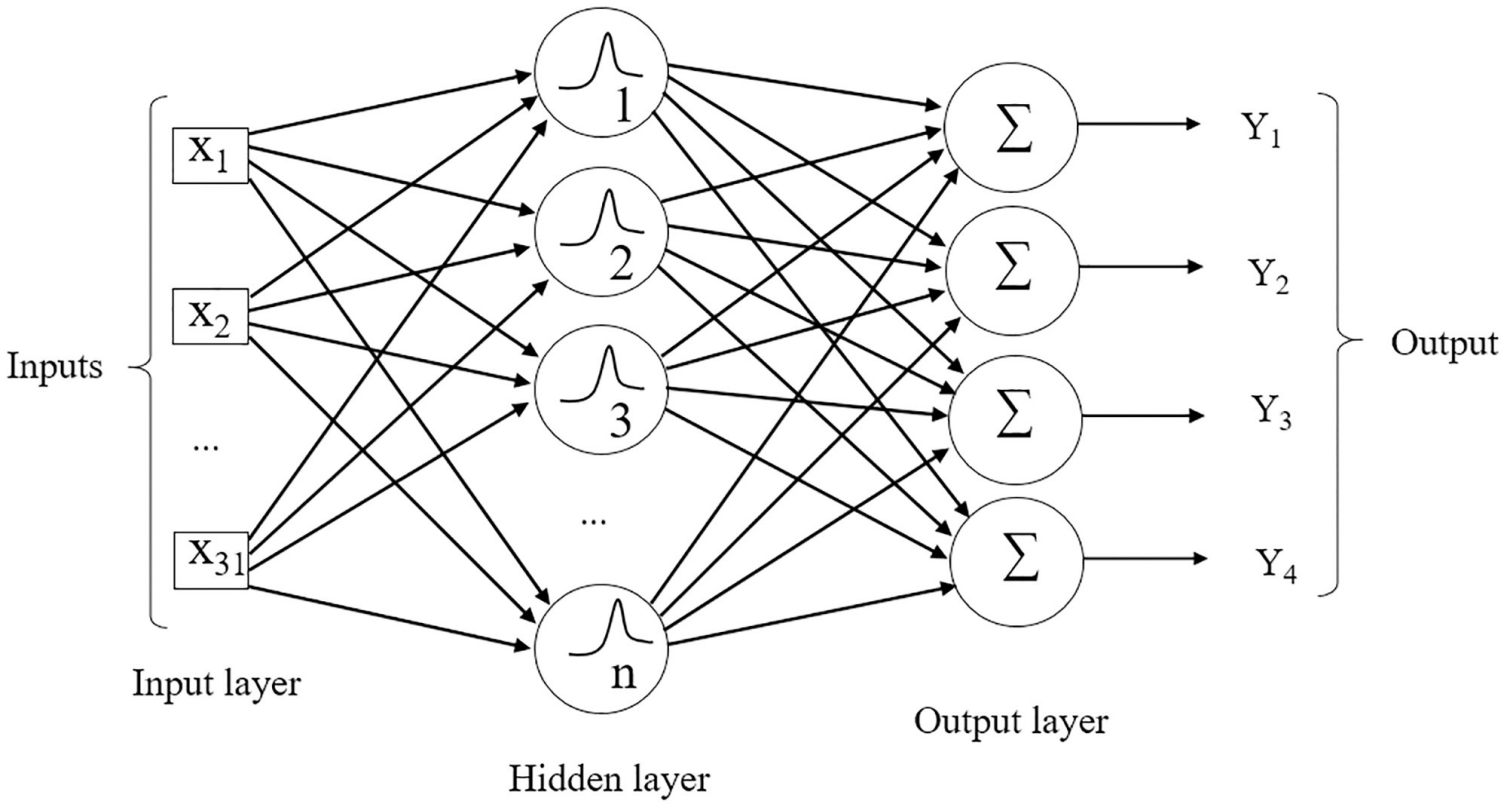
Example of a feedforward RBF network. Inputs X_1_ to X_31_ in the input layer refer to the 31 traits evaluated. A hidden layer with radius ranging from 5 to 15 (r = 1, …, 15) and number of neurons (n) ranging from 10 to 50 (n = 1, …, 50). At the output, the network returns a matrix with values of 1 and 0 of dimension *i* x *j*, where *i* is the number of observations and *j* the number of environments.

The architecture of the RBF is of the feedforward type with one input layer, one intermediate layer, and one output layer [57]. For the stopping criterion, the mean quadratic error equal to 0 was adopted to maximize the network performance, allowing to obtain the smallest possible error. As performed for MLP, Nguyen-Widrow algorithm (INITNW) [53] was utilized to initialize the weights, dataset was normalized, so that the maximum is 1 and the minimum is 0 and preliminary analyzes for RBF were also carried out, where lower error rates were found in the network for the number of neurons ranging from 10 to 50 in the hidden layer, with a radius ranging from 5 to 15. The model used in the Radial Basis Function network is shown in Fig 2.

#### Classification tree

The purpose of analysis using tree-building algorithms is to determine a set of logical conditions (split) ’if-then’ that allows for accurate classification or prediction of cases, known as classification and regression tree models (C&RT) [28]. Classification trees (CT) can be considered as a collection of rules that allow separate sets of resources to be linked to a common class [58]. The tree resembles a graph consisting of a root node from which at least two branches emerge, which leads to lower nodes (child nodes). A tree-structured decision space is estimated by recursively splitting the data at each node based on a statistical test that increases the homogeneity of the training data in the resulting descendant nodes [59]. Each node is assigned a class description, and each branch refers to a decision rule, that is, a condition related to the resources of the input data set and that describes the case when each branch is chosen [28, 58].

The mathematical background used for the classification tree was defined according to Krasteva et al. [28]:

There are *n* observations in a parent node *P* and there are *J* classes labeled as *P*_1_, *P*_2_, *P*_3_, …, *P*_*j*_. Let *n*_*j*_ be the number of individuals in the class *j*, where *j* is evaluated environment. The relative proportion $\frac{n_{j}}{n}$ of class *j* individuals in the node is denoted by *p*_*j*_. Each binary split *s*_*i*_ produces two child nodes–left (*L*), which contains *n*_*L*_ individuals and right (*R*) with *n*_*R*_ individuals, such that *n*_*L*_ + *n*_*R*_ = *n*. The child nodes contain the relative proportions $P_{L}=\frac{n_{L}}{n}$ and $P_{R}=\frac{n_{R}}{n}$. The relative proportions of class *j* individuals in the child nodes are denoted by *P*_*jL*_ and *P*_*jR*_. The notation *i*(*p*) is further used as a generic notation of impurity, formulated in the present study, based on entropy or maximum deviation reduction to minimize the mean square error. Entropy or maximum deviation reduction is given as:

$$i_{E}(p)=-\sum_{j=1}^{j}p_{j}logp_{j}$$

When the entropy of a node is zero, *p*_*j*_ = 1 class *j*, then the node is said to be pure, since it contains individuals of only one class (environment). When the entropy is maximized, *p*_*j*_ is uniform, then the node is least pure because it contains equal proportions of individuals from each environment. The “tree” and “ISLR” R packages were used for this procedure [60, 61].

#### Bagging

The basic idea of Bootstrap aggregation or Bagging is to create several similar data sets by resampling (bootstrapping) and regression trees that are performed without pruning and averaging [39, 62]. Some prediction method is applied to each bootstrap sample, and then the results are combined, by averaging for regression and simple voting for classification, to obtain the overall prediction. The portion of the data drawn into the sample in replication is known as the ‘‘in-bag” data, whereas the portion not drawn is the ‘‘out-of-bag” data. According to Prasad et al. [39], "out-of-bag" data are not used to build or prune any tree, but provide better estimates of node error and other generalization errors for bagged predictors. Besides that, the instability created by the different trees analyzed separately generates a reduction in variance and an improved average of the results.

Suppose a training data model *Z* = {(*x*_1_, *y*_1_), (*x*_2_, *y*_2_), …, (*x*_*N*_, *y*_*N*_)}, obtaining the prediction $\hat{f}(x)$ at input *x*. Bagging averages these predictions over a collection of bootstrap samples, thereby reducing its variance. For each bootstrap sample Z*^b^, *b* = 1, 2, …, *B*, we fit our model, giving prediction ${\hat{f}}^{*b}(x)$. In the words, bagging consists of obtaining one number B of samples with replacement (size equal to N) of the data set, obtaining models *f*_1_(*x*), *f*_2_(*x*), …, *f*_*B*_(*x*)). Each model is used as an individual classifier. A new individual will be allocated to the most common class among the predictions of the individual B classifiers [38]. The bagging estimate is defined by [2]:

$${\hat{f}}_{bag}(x)=\frac{1}{B}\sum_{b=1}^{B}{\hat{f}}^{*b}(x)$$

Denote by $\hat{\rho }$ the empirical distribution putting the equal probability $\frac{1}{N}$ on each of the data points (*x*_*i*_, *y*_*i*_). The “true” bagging estimate is defined by $E_{\hat{\rho }}{\hat{f}}^{*}(x)$, where $Z^{*}=(x_{1}^{*},y_{1}^{*}),(x_{2}^{*},y_{2}^{*}),\ldots ,(x_{N}^{*},y_{N}^{*})\sim \hat{\rho }$.

The number of trees to grow was fixed as 500. To carry the Bagging, the “randomForest” R package was used [63].

#### Random forest

Random forest [64] is similar to Bagging, in that bootstrap samples are drawn to construct multiple trees; the difference is that each tree is grown with a randomized subset of predictors. The number of predictors used to find the best split at each node is a randomly chosen subset of the total number of predictors. As with Bagging, the trees are grown to maximum size without pruning, and aggregation is by averaging the trees. In the Random Forest model, out-of-bag samples are used as internal validation from which the out-of-bag error is computed. Because a large number of trees are grown, there is limited generalization error, which means that no overfitting is possible [39]. The random forest results in a process of eliminating the correlation between the trees generated, further improving the accuracy of forecasts. However, it uses a smaller number of predictive traits in each division concerning Bagging [20, 38]. This is achieved in the tree-growing process through random selection of the input variables [58].

To make a classification of new point x:

Let ${\hat{C}}_{b}(x)$ be the class prediction of the bth random-forest tree. Then ${\hat{C}}_{rf}^{B}(x)=marjority\;vote\;{\left\{{\hat{C}}_{b}(x)\right\}}_{1}^{B}$.

The number of trees to grow were fixed as 500 and number of individuals selected at each tree node was given $m=\sqrt{p}$, *p* being the number of variables. The “randomForest” R package were used for this procedure [63].

#### Boosting

In Boosting, bias is reduced by repeatedly readjusting the weights of the training samples, by focusing on ‘‘difficult” examples from previous samples [39]. Boosting creates trees sequentially using information from previous trees, unlike Bagging which creates multiple independent trees [38]. The Boosting classifier has the form $H(x)=\sum_{t}^{\propto_{t}}h_{t}(x)$, which seeks to minimize a loss function by optimizing the scalar t (importance attributed to *h*_*t*_(*x*)) and the individual classifier *h*_*t*_(*x*) at each iteration [65]. The individual classifiers *h*_*t*_(*x*) have low classificatory power, but when used together *H*(*x*), they present good results [38, 66]. The number of trees to grow was fixed as 500, with interaction depth equal to 2 and rate learning—shrinkage equal to 1%, and Multinomial distribution was adopted. To carry the Boosting, the “gbm” R package was used [67].

In Table 2 we highlight the particularities of the method used in terms of the type of learning, the applicability, and the limits of each technique.

**Table 2:** Summary of the particularities of the methods used to classify individuals in multi-environments, with their respective learning, types, applications, and limits.

Methods	Learning	Types	Applications	Limits
Fisher	Statistical	Supervised	Classificatory	Linear
Anderson	Statistical	Supervised	Classificatory	Linear
Classification Tree	Machine learning	Supervised	Classificatory and predictive	Linear and non-linear
Bagging	Machine learning	Supervised	Classificatory and predictive	Linear and non-linear
Random Forest	Machine learning	Supervised	Classificatory and predictive	Linear and non-linear
Boosting	Machine learning	Supervised	Classificatory and predictive	Linear and non-linear
Multilayer Perceptron	Machine learning	Supervised	Classificatory and predictive	Linear and non-linear
Radial Basis Function	Machine learning	Hybrid (unsupervised and supervised)	Classificatory and predictive	Linear and non-linear

### Training and test population

**Fig 3:**
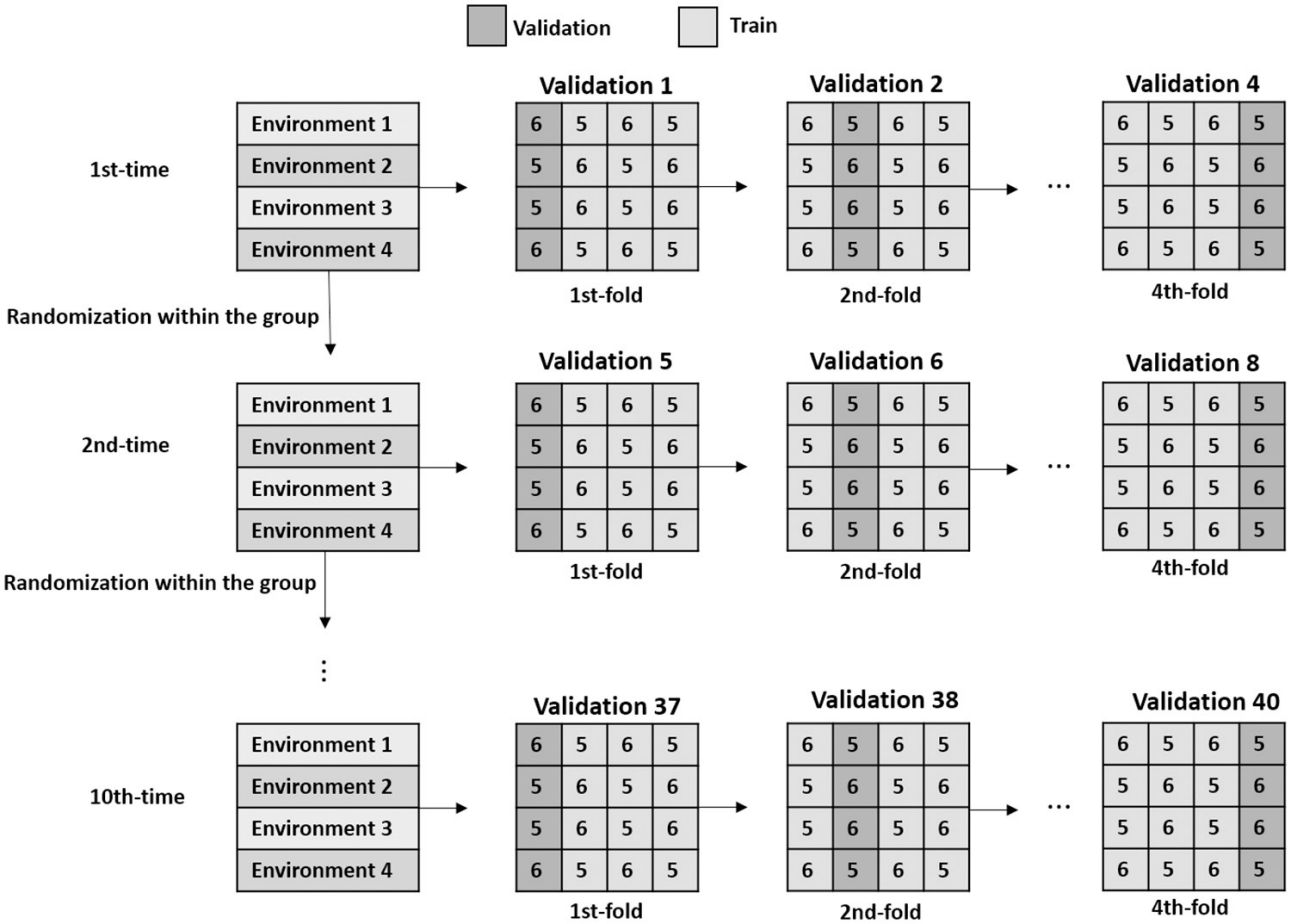
K-fold cross-validation process, taking samples of training and testing. The number inside the box represents the proportion of individuals from each environment in the test group.

The performance of those methods has been verified using the testing database. Also, k-fold cross-validation procedure was used to mitigate the bias caused by the random selection of the data [68, 69]. The total data set was divided into 4 folds, each fold contained approximately 22 individuals so that all environment was represented in the test (Fig 3). To maintain the proportionality of the individuals of each environment within the training and test groups, since the division of 22 by 4 does not result in an integer, in the validation group there could be 5 or 6 individuals from each population, so that at the end of each cycle, with 4 folds, each population will have an average of 5.5 individuals for validation. Every iteration, three folds were used for training and one-fold to test the method. This procedure was repeated 10 times after randomization of the individuals within each group, followed by a new division of the 4-folds. and with cross-validation divided the data set into four-folds. Three folds were used for training and one to test the method. The individuals present in the test set were not previously seen by the methods during the training phase.

### Apparent Error Rate (AER)

The apparent error rate (AER) measures the efficiency of the methods to classify individuals correctly in the previously established environments. AER was determined by the ratio between the number of wrong classifications and the total number of classifications, according to Cruz et al. [51]:

$$AER(\%)=\frac{1}{N}\sum_{l=1}^{4}m_{j},$$

where *m*_*j*_ is the number of genotypes from the environment *A*_*l*_ that were classified in another environment *A*_*l*′_, where *l*′ = *l* and *l* = 1, 2, …, 4 environments; considering: $N=\sum_{l=1}^{22}n_{l}$, where *n*_*l*_ is the number of genotypes related to the *A*_*l*_ environment.

Final AER for each method was given by the average of the 10 replicates and for the comparison of efficiency and definition of the methodology with the best classification power was performed the Scott-Knott test, at a 5% probability.

### Importance of traits

The calculation of the relative importance of the traits was carried out according to the specific technique of the methodology (s) with the best classificatory power. The importance analysis of traits was performed through the technique indicated by Fischer [70]. For each input trait, the network forecast is calculated after setting all weights (only) of this trait to zero, to obtain a value for apparent error rate (AER) of the modified network.

Another technique utilized to obtain the importance of traits, not yet studied in other articles and proposed in the present work, is the randomization of each trait at the input of the network. In this way, the randomized trait loses its relationship with the output trait. If this randomization results in a reduction in the efficiency of the network, it implies that the randomized trait is important in printing the final result, according to the magnitude of the reduction in efficiency resulting after its randomization. This proposal is in the Genes software [71].

Each input trait the apparent error rate (%) of the modified network prevision (*AER*_*mod*_) can be compared with the apparent error rate (%) of the complete network prevision (*AER*_*com*_), to obtain the magnitude of the effect of the input trait for each test set. The magnitude of the effect of the input trait j was given by the sum of the magnitudes in the test sets performed ((${AER}_{j}=\sum_{i}^{k}{\;({AER}_{mod(j)}\;-\;{AER}_{com})}_{i}$). To facilitate interpretation, the *AER*_*j*_ values were transformed to a percentage scale, representing the relative importance (*RI*) of each trait, according to the equation below:

$${RI}_{j}(\%)=\frac{\sum_{i}^{3}{\;({AER}_{mod(j)}\;-\;{AER}_{com})}_{i}}{\sum_{j}^{31}\sum_{i}^{3}{\left({AER}_{mod(j)}\;-\;{AER}_{com}\right)}_{i}\;}\;x\;100$$

where *RI*_*j*_ is the relative importance of trait j in percentage; *AER*_*mod* (*j*)_ is the apparent error rate of the model modified with the randomization of trait j; *AER*_*com*_ is the apparent error rate of the complete model; *i* = number of repetitions (test set), ranging from 1 to 10; and j = number of traits, ranging from 1 to 31.

The linear discriminant analysis by Fischer and Anderson and the methods of Classification Tree, Boosting, Bagging, and Random Forest were performed by the Genes software in integration with the R software [71, 72]. And similarly, the MLP and RBF models were performed by the Genes software in integration with the Matlab software [71, 73].

## Results and discussion

The attributes clean cup, sweetness, and uniformity showed no variance and reached a maximum score (10 points) for all genotypes in different environments. Similar results were found by Gamonal et al. [74], when evaluating four genotypes at different altitudes, despite finding variance, also found no significant differences for these same attributes. Thus, these sensory attributes were discarded from later analyzes to discriminate environments.

### Pattern recognition

**Fig 4:**
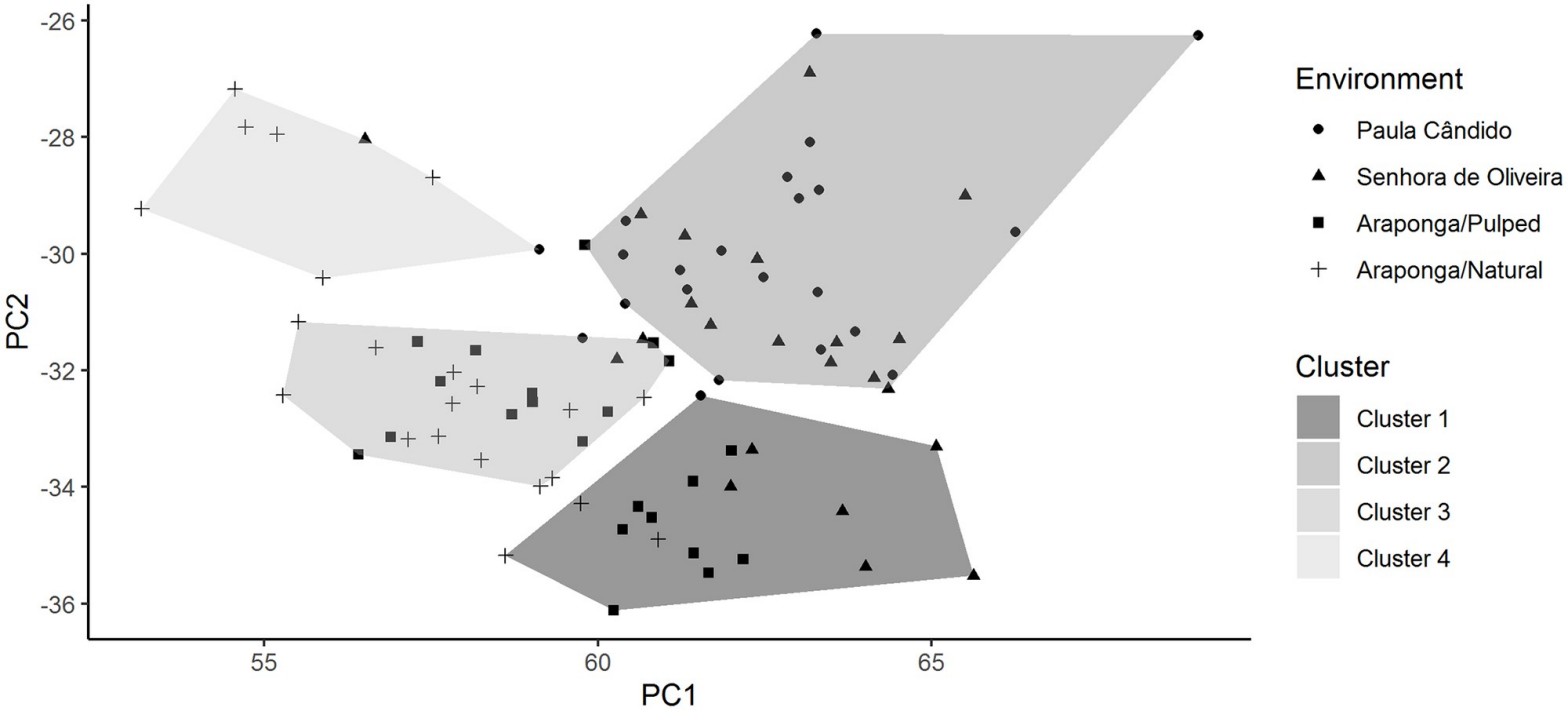
Graphic dispersion of traits evaluated in *Coffea arabica* experiments about the principal components analysis. Cluster 1- Paula Cândido/Pulped; Cluster 2- Senhora de Oliveira/Pulped; Cluster 3- Araponga/Pulped; Cluster 4- Araponga/Natural; PC1- principal component 1; PC2- principal component 2.

The clusters formed by the pattern recognition analysis performed by the Kohonen self-organizing maps (KSOM) method were not efficient in revealing the grouping of the individuals in their respective assessment environments (Fig 4). Thus, even using an artificial intelligence methodology, such as the KSOM method, in the multivariate context, it was not possible to group individuals according to the expected clustering pattern, that is, the problem in question is not linearly separable.

### Classification of environments

In Table 3 is showed the count of individuals classification of the test set. Fisher and Anderson discriminant functions also had difficulty in correctly distinguishing individuals in their environments. Especially in more similar environments, such as Araponga—Pulped (3) and Araponga—Natural (4). These results proved to be not possible to classify the coffee samples produced in their respective assessment environments to discriminate them linearly.

**Table 3:** Confusion matrix of the classification of individuals in four groups of the Fisher and Anderson discriminant functions, according to the multi-environment genotypes evaluated in the region of Matas de Minas in Minas Gerais.

Environment	Fisher					Anderson				
	Groups				Total	Groups				Total
	1	2	3	4		1	2	3	4	
**Paula Cândido—Pulped**	4.400	0.150	0.575	0.375	5.500	4.350	0.075	0.700	0.375	5.500
**Senhora de Oliveira–Pulped**	0.225	5.125	0.075	0.075	5.500	0.200	5.050	0.225	0.025	5.500
**Araponga–Pulped**	0.150	0.025	3.625	1.700	5.500	0.150	0.000	3.700	1.650	5.500
**Araponga–Natural**	0.100	0.150	1.650	3.600	5.500	0.100	0.050	1.800	3.550	5.500
**Total**	4.875	5.450	5.925	5.750	22.000	4.800	5.175	6.425	5.600	22.000

James et al. [20] had already pointed out that the linear discriminant analysis is only fully functional for linearly separable problems. The absence of a known distribution of traits in a data set is one of the peculiarities that can hinder analysis in a stochastic methodology, as in the case of statistical methodologies. This can be overcome by using artificial intelligence methodologies, in which such assumptions are not necessary and their results depend on learning [75].

**Fig 5:**
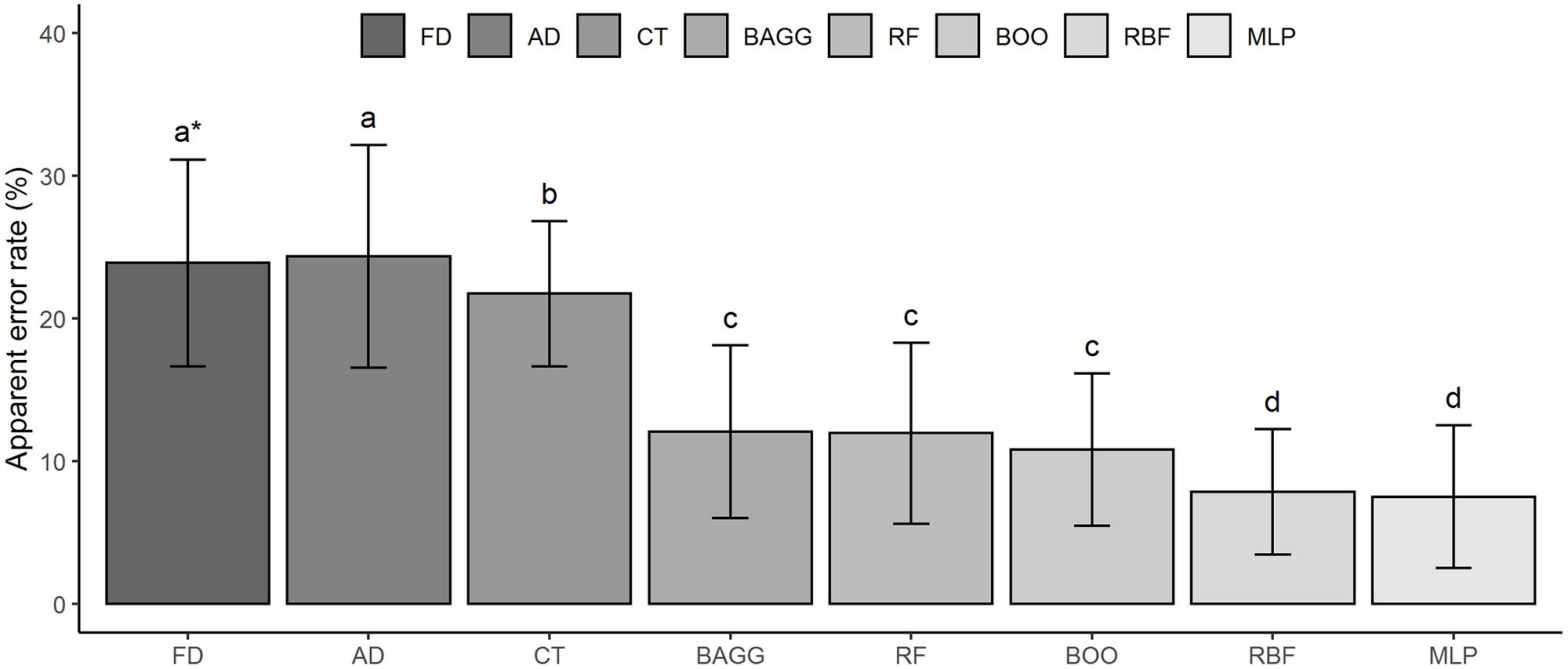
Apparent Error Rates (AER) in the test sets according to the classification methods. FD = Fisher Discriminant, AD = Anderson Discriminant, CT = Classification Tree, BAG = Bagging, RF = Random Forest, BOO = Boosting, RBF = Radial Basis Function, MLP = Multilayer Perceptron. *Letters followed by the same letter do not differ by the Scott and Knott test at a 5% probability level.

Fischer and Anderson discriminant analyzes were compared with machine learning models, according to the apparent error rates for the test data set, considering the 10 repeats (Fig 5). The discriminant analyzes of Fischer (23.85%) and Anderson (24.34%), Classification tree (21.73%), Bagging (12.06%), Random forest (11.95%) and Boosting (10.81%) presented higher error rates (Fig 5). The RBF (7.85%) and the MLP (7.50%) presented means of apparent error rate different from the other methods by the Scott and Knott test, therefore, they showed better classificatory power than the other methodologies. Besides, it was possible to observe that all models based on artificial intelligence showed greater accuracy to statistical analyzes since they presented less amplitude of the deviation values about the mean obtained from the test sets. Thus, the artificial intelligence methods were less affected by the changes caused by the individuals between the test sets.

Based on the confusion matrix presented in Table 4, it was found that by artificial neural networks (RBF and MLP) individuals from the environments of Paula Cândido and Senhora de Oliveira can be almost totally discriminated against without confusion. Thus, the errors obtained by the ANNs were essentially due to the erroneous classification of individuals among the environments of Araponga.

**Table 4:** Confusion matrix of the classification of individuals in four groups of the Radial Basis Function (RBF) and Multilayer Perceptron (MLP), according to the multi-environment genotypes evaluated in the region of Matas de Minas in Minas Gerais.

Environment	RBF					MLP					
	Groups				Total	Groups				Total
	1	2	3	4		1	2	3	4	
**Paula Cândido—Pulped**	5.375	0.050	0.075	0.000	5.500	5.375	0.125	0.000	0.000	5.500
**Senhora de Oliveira—Pulped**	0.000	5.450	0.050	0.000	5.500	0.000	5.500	0.000	0.000	5.500
**Araponga—Pulped**	0.000	0.000	4.375	1.125	5.500	0.075	0.000	4.400	1.025	5.500
**Araponga—Natural**	0.025	0.000	0.425	5.050	5.500	0.025	0.000	0.400	5.075	5.500
**Total**	5.400	5.500	4.925	6.175	22.000	5.475	5.625	4.800	6.100	22.000

As the individuals from the Araponga—Natural and Araponga—Pulped environments present redundant values for the pre-harvest traits, the results found by the confusion matrix (Table 4), reinforces that the ANNs is a good alternative for classification problems involving individuals with high similarity.

Unlike the results obtained in this study, when using neural network modeling and Fisher discriminant analysis for selection among sugarcane families, Peternelli et al. [34] found that the networks presented results similar to the statistical analysis. The results found by these authors indicated that the problem evaluated by them was linearly separable, and therefore the results were similar for the methodologies. For situations in which the data show multivariate Poisson distribution and homogeneity of covariance matrices, Fisher discriminant function and Artificial Neural Networks have lower values of AER compared to other methods [24].

Studies carried out on the diversity of papaya accessions (*Carica papaya* L) [76] and study with simulated genotypic data from 10 populations in Hardy-Weinberg equilibrium [33], showed that ANNs were better classifiers about conventional discriminant analysis, corroborating the results obtained in this study. These results can be explicated to situations in which the data have multivariate normality and homoscedasticity of covariance matrices, the ANNs feature better results [24]. It is important to mention that for the correct choice of the methodology to be applied, the variety of information and the particularities of each biological material must be taken into account, in addition to performing a careful analysis as performed in this study.

### Importance of traits

The analysis of the importance of the variables was evaluated only for the PMC and RBF models since they presented better results. For this, two strategies were considered based on the results of neural networks. The first was indicated by Fischer [70], each explanatory trait has its weight reset to zero, and subsequently, the apparent error rate of the modified network is calculated. Another technique used to obtain the importance of the traits, not yet evaluated in other articles and proposed in this work, is the randomization of the values of each explanatory trait. In this technique, the explanatory trait that has its values randomized would lose its relationship with the predictor trait. If the randomization of the explanatory trait reduces the efficiency of the modified network (increase in the AER value), the importance of this trait is in accord with the magnitude of the reduction in network efficiency. This method proves to be more interesting than the proposed by Fischer [70], because, with the randomization, the explanatory trait will still present values within a biological range, unlike when the values of the trait are zeroed, thus being a less drastic form disturbance of the trait.

**Fig 6:**
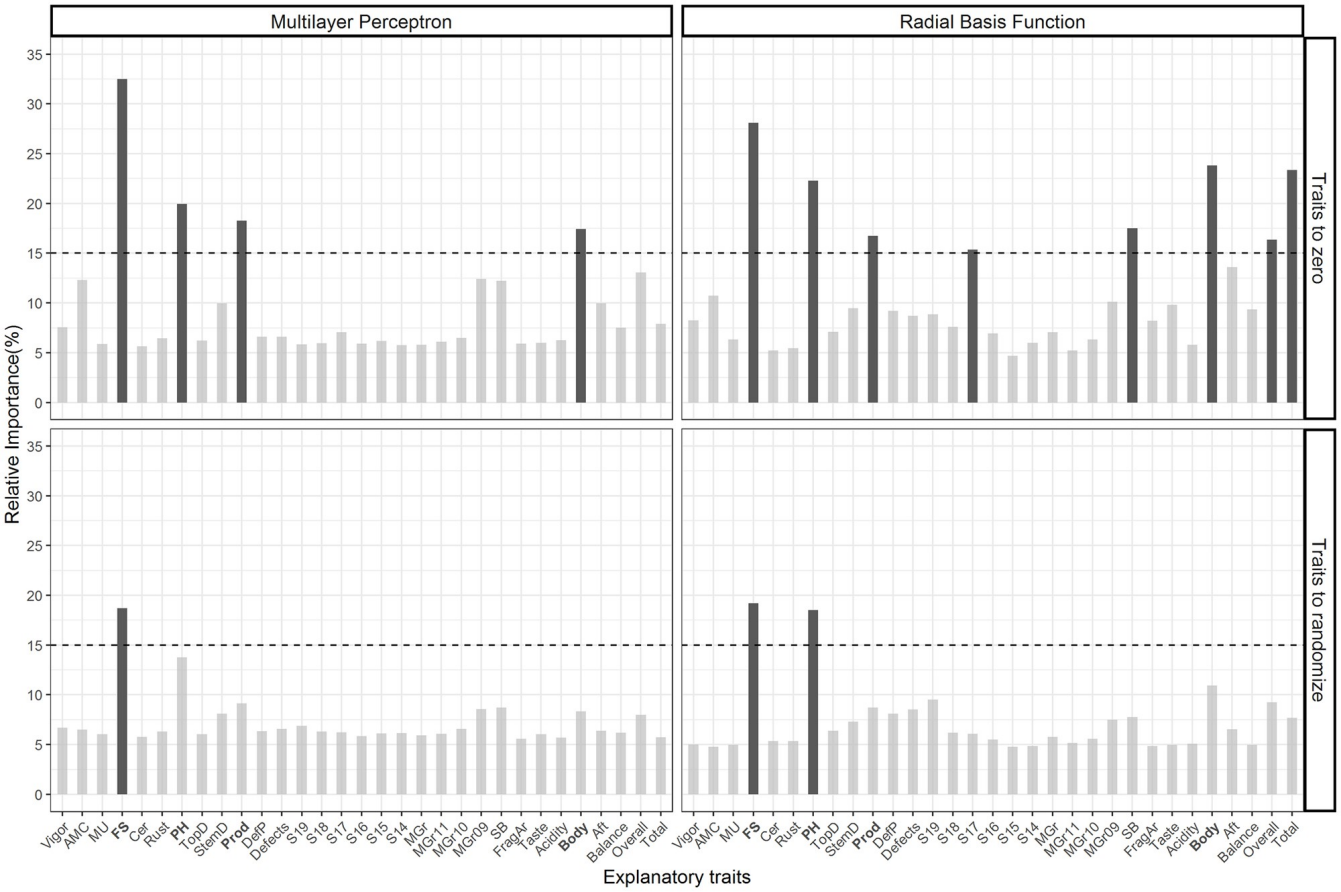
Percentage of the relative importance of the 31 explanatory traits for classifying individuals according to multi-environment by the techniques of making trait value to zero and randomizing the trait value by the ANNs (Multilayer Perceptron and Radial Basis Function). The dashed line is the cutting line equal to 15%. In bold: traits with average relative importance greater than 15%.

A cut-off point was created on the axis of relative importance, where only the traits that presented values of relative importance greater than 15%, on average in the four quadrants of Fig 6, were determined as important traits. Thus, there is greater certainty about the real importance of the trait.

The phenotypic traits: plant height, mature fruit size, and production, together with the sensory trait body, they were mainly responsible for the discrimination of environments (Fig 6). These phenotypic traits were fundamental for the discrimination of the production sites, observed by the different average values in the three locations (Table 5). Body sensory, on the other hand, proved to be of great importance for the classification of coffees in different post-harvest processing.

**Table 5:** Averages and standard deviations from traits assessed in different environments and post-harvest processing.

Traits*	Paula Cândido	Senhora de Oliveira	Araponga Pulped	Araponga Natural
Vigor	8.549 (±0.309)	7.849 (±0.339)	7.831 (±0.594)	7.831 (±0.594)
AMC	2.923 (±0.173)	2.827 (±0.161)	3.200 (±0.336)	3.200 (±0.336)
MU	2.842 (±0.207)	3.045 (±0.210)	2.568 (±0.402)	2.568 (±0.402)
FS	1.995 (±0.026)	2.069 (±0.087)	3.182 (±0.262)	3.182 (±0.262)
Cer	1.911 (±0.182)	1.833 (±0.331)	1.600 (±0.509)	1.600 (±0.509)
Rust	1.761 (±0.619)	2.202 (±0.447)	2.168 (±1.214)	2.168 (±1.214)
PH (m)	1.831 (±0.113)	2.389 (±0.132)	1.319 (±0.075)	1.319 (±0.075)
TopD (m)	1.316 (±0.085)	1.431 (±0.086)	1.173 (±0.064)	1.173 (±0.064)
StemD (cm)	4.392 (±0.233)	5.654 (±0.477)	3.529 (±0.172)	3.529 (±0.172)
Prod (bags ha^-1^)	66.943 (±15.178)	21.641 (±6.144)	57.715 (±8.875)	57.715 (±8.875)
DefP (%)	22.000 (±5.273)	14.364 (±4.306)	13.818 (±2.620)	11.500 (±2.818)
Defects	188.955 (±46.847)	123.773 (±37.116)	118.909 (±21.545)	99.045 (±24.149)
S19 (%)	8.773 (±5.459)	5.364 (±3.884)	4.000 (±3.273)	5.227 (±3.723)
S18 (%)	16.727 (±5.636)	14.727 (±6.702)	16.727 (±7.157)	16.773 (±6.661)
S17 (%)	22.955 (±5.058)	25.318 (±4.471)	30.364 (±4.273)	29.909 (±2.992)
S16 (%)	17.091 (±5.934)	21.727 (±6.050)	22.727 (±6.273)	21.636 (±6.066)
S15 (%)	7.727 (±2.678)	9.864 (±4.917)	7.591 (±3.169)	7.364 (±3.579)
S14 (%)	4.136 (±0.731)	3.636 (±1.388)	3.545 (±1.140)	3.545 (±1.008)
MGr (%)	18.227 (±3.814)	15.727 (±3.471)	11.045 (±2.240)	10.636 (±1.909)
MGr11 (%)	7.045 (±1.971)	5.500 (±1.455)	3.364 (±1.066)	3.318 (±0.674)
MGr10 (%)	8.136 (±2.124)	7.227 (±2.037)	5.273 (±1.529)	5.636 (±1.182)
MGr09 (%)	3.045 (±0.607)	3.000 (±0.818)	2.409 (±0.682)	1.773 (±0.583)
SB (%)	4.364 (±1.033)	4.091 (±1.281)	4.000 (±1.091)	4.833 (±1.000)
FragAr	7.788 (±0.231)	7.788 (±0.187)	7.773 (±0.183)	7.508 (±0.268)
Taste	7.894 (±0.211)	7.955 (±0.218)	7.894 (±0.190)	7.523 (±0.343)
Acidity	7.568 (±0.123)	7.712 (±0.281)	7.674 (±0.267)	7.386 (±0.205)
Body	7.947 (±0.092)	7.917 (±0.136)	7.902 (±0.143)	7.561 (±0.213)
Aft	7.871 (±0.198)	7.864 (±0.285)	7.894 (±0.230)	7.439 (±0.354)
Balance	7.576 (±0.171)	7.705 (±0.238)	7.727 (±0.227)	7.455 (±0.116)
Overall	7.629 (±0.186)	7.742 (±0.244)	7.750 (±0.242)	7.386 (±0.185)
Total	84.180 (±1.07)	84.680 (±1.41)	84.610 (±1.14)	82.260 (±1.55)

*Vegetative vigor was according to a score that ranged from 1 to 10; Fruits maturation cycle (AMC), Severity of cercosporiosis (Cer) and rust (Rust) were according to a score that ranged from 1 to 4; Fruits maturity uniformity (MU) was according to a score that ranged from 1 to 5; Mature fruit size (FS) was according to a score that ranged from 1 to 3; Defects was measured number of defective grains per 300g of sample; Sensory attributes scores were given, in the range of 0 to 10 points; Total score were given, in the range of 0 to 100 points.

Pulped coffee had the highest score for the body attribute (Table 5). Besides, we can highlight that pulped coffee obtained higher scores for sensory traits and in high altitude environments, the behavior quality was better (Table 5), as also found by Bote and Vos [77] and Barbosa et al. [78] in univariate sensory analysis.

Mature fruit average size increased as the altitude of the production site was higher. In this way, Araponga presented larger fruits, followed by the fruits produced in Senhora de Oliveira, and finally, the Paula Cândido, a site of lower altitude (Table 5). The altitude influences the coffee cycle, as well as the accumulation of Ca, Mg and S in the fruits due to the higher fruit/leaf competition in low altitude environments [79]. On the other hand, at higher altitudes, the tendency is for the same genotype to produce bigger fruits, as fruit maturation will be delayed, which provides the possibility of further expansion, due to the accumulation of photoassimilates [80, 81].

In Paula Cândido the height of the plants was 1.831m (± 0.139). The greater density of plants can influence their growth due to phototropism [82]. This could justify a higher average plant height in this environment since Paula Cândido is the environment with the highest density of plants (Table 1). However, taller plants were found in Senhora de Oliveira (2.389m ± 0.165). This result can be justified by the difference in the planting date, since it was carried out first in Senhora de Oliveira (2009), followed by Paula Cândido (2012) and lastly in Araponga (2013).

Although Paula Cândido had plants with smaller heights, this was the place that presented the highest bean mean production (66.94 bg ha^-1^), followed by the productivity in Araponga (57.71 bg ha^-1^) and Senhora de Oliveira (21.64 bg ha^-1^) (Table 5). These results do not corroborate with those described in the literature, where several authors observed the presence of positive correlations between plant height and productivity [82, 83].

The morpho-agronomic traits maturity uniformity, top diameter, cercosporiosis, and rust severity presented a little contribution to the process of classifying individuals according to the different production sites (Fig 6). This shows that these traits have little influence on the differentiation of local factors. With these results, it was found that the sites did not present specific environmental that favored the incidence of diseases and neither did the response of plants to them, also observed by the non-differentiation of vegetative vigor. Other traits of low importance were the percentage of mocas grains (MGRr, MGRr10, and MGRr11) and classification of flat grains in sieves (S14, S15, S16, and S18), which did not allow to discriminating production sites based only on these traits (Fig 6).

Similarly, the sensory traits acidity and fragrance/aroma also made little contribution to the process of classifying individuals in environments (Fig 6). These results indicate that acidity and fragrance/aroma score can be a characteristic of coffees produced in the Matas de Minas region and with little response according to post-harvest processing. On the other hand, no trait showed null importance for the two techniques used to calculate the relative importance. This can be justified by the fact that all traits participated in a way for the classification of individuals, even if only in just one repetition. Thus, even if there are traits that make little contribution to the classification process, all the traits available for analysis of importance of traits must be considered.

## Conclusions

Machine learning models are able to discriminate coffee samples submitted to different environments and post-harvest processing more efficiently than linear statistical methods. However, among the various models used, the artificial neural networks of Radial Basis Function and Multilayer Perceptron presented a lower apparent error rate average (7.85 and 7.50%, respectively) and, thus, proved to be the most efficient.

Finally, the analyzes demonstrated the presence of environmental and post-harvest processing effects in the expression of traits, which reflects in a multivariate way in the productive traits of the coffee tree and its final product. By the techniques of the importance of traits making trait value to zero and randomizing the trait value, based in artificial neural networks, the main traits responsible for the differentiation of production sites were: fruit size, plant height, and production; in addition to the sensory trait “Body”, which was fundamental to discriminate the type of post-harvest processing.
